# Supplementary material for: Parametrically driving a quantum oscillator into exceptionality
Source: Sci Rep. 2023 Jul 7;13:11004. doi: 10.1038/s41598-023-37964-7 (PMC10329046; doi:10.1038/s41598-023-37964-7)
Supplement: Supplementary file 1 — Supplementary Information. [file 41598_2023_37964_MOESM1_ESM.pdf]

Supplementary Information:
Parametrically driving a quantum oscillator into exceptionality

C. A. Downing[1,*] and A. Vidiella-Barranco[2]

[1]*Department of Physics and Astronomy, University of Exeter, Exeter EX4 4QL, United Kingdom*
[2]*Gleb Wataghin Institute of Physics, University of Campinas - UNICAMP, 13083-859, Campinas, SP, Brazil*

In this Supplementary Information, we lay out the quantum optical theory underpinning the results presented in the main text regarding a parametrically driven-dissipative quantum oscillator. We also discuss the effects of nonzero temperature of the thermal bath.

CONTENTS

I. Hamiltonian 2

II. Quantum master equation 3

III. First-order coherence 5

IV. Second-order coherence 6

V. Optical spectrum 6
A. Doublet regime 7
B. Singlet regime 7

VI. Second and fourth moments 7

VII. Interactions 9
A. Liouvillian 10
B. Correlators 11
C. Semi-classical approximation 12

VIII. Nonzero temperature 13
A. Robustness of the exceptional point 13
B. Some auxiliary results 14

* c.a.downing@exeter.ac.uk

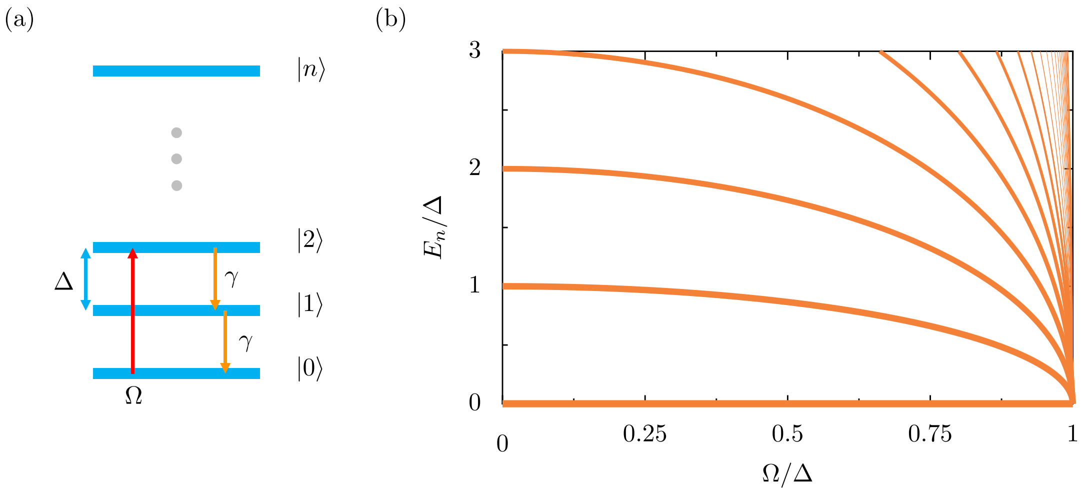

FIG. S1. **Energy levels of the parametric driven-dissipative oscillator.** Panel (a): a sketch of the infinite and bosonic energy ladder of the system, including the detuning Δ (cyan arrow), the parametric driving amplitude Ω (red arrow), and the damping decay rate γ (orange arrows). The number states $|n\rangle$ (where $n = 0, 1, 2, ...$) are noted alongside the energy ladder. Panel (b): The lowest eigenenergies E_n as a function of Ω, where both quantities are given in units of Δ [cf. Eq. (S7)]. These eigenenergies are associated with certain eigenstates, which are squeezed number states, denoted by $|n, \phi\rangle$ [cf. Eq. (S7)].

I. HAMILTONIAN

The Hamiltonian $\hat{\mathcal{H}}$ of the parametric driven-dissipative oscillator discussed in the main text [cf. the sketch in Fig. S1 (a)] contains two energetic contributions, and it reads

$$\hat{\mathcal{H}} = \hat{\mathcal{H}}_0 + \hat{\mathcal{H}}_{\mathrm{D}}, \tag{S1}$$

where the energy levels (equally separated by the frequency ω_0) of the quantum harmonic oscillator are contained within $\hat{\mathcal{H}}_0$, while the parametric driving term is $\hat{\mathcal{H}}_{\mathrm{D}}$. These Hamiltonian pieces separately read (where we take $\hbar = 1$ throughout)

$$\hat{\mathcal{H}}_0 = \omega_0 b^\dagger b, \qquad \hat{\mathcal{H}}_{\mathrm{D}} = \frac{\Omega \mathrm{e}^{\mathrm{i}\theta}}{2} \mathrm{e}^{-2\mathrm{i}\omega_{\mathrm{D}} t} b^\dagger b^\dagger + \frac{\Omega \mathrm{e}^{-\mathrm{i}\theta}}{2} \mathrm{e}^{2\mathrm{i}\omega_{\mathrm{D}} t} bb, \tag{S2}$$

where the driving amplitude $\Omega \geq 0$, the driving phase $-\pi \leq \theta \leq \pi$ and the driving frequency ω_{D} have entered the model. The bosonic creation and annihilation operators $b^\dagger$ and b satisfy the commutation relation $[b, b^\dagger] = 1$. Clearly, the parametric drive necessarily links the zero and two-excitation sectors (for example), as is implied by the red arrow in Fig. S1 (a). Moving into a rotating frame of reference with the transformation U leads to the general rotated Hamiltonian $\hat{H}$, given by

$$\hat{H} = U \hat{\mathcal{H}} U^\dagger + \mathrm{i} \left(\partial_t U\right) U^\dagger, \qquad U = \mathrm{e}^{\mathrm{i}\omega_{\mathrm{D}} t \; b^\dagger b}, \tag{S3}$$

which for the considered case of Eq. (S1) leads to the specific form of the overall governing Hamiltonian [cf. Eq. (1) from the main text]

$$\hat{H} = (\omega_0 - \omega_{\mathrm{D}}) \, b^\dagger b + \frac{\Omega \mathrm{e}^{\mathrm{i}\theta}}{2} b^\dagger b^\dagger + \frac{\Omega \mathrm{e}^{-\mathrm{i}\theta}}{2} bb. \tag{S4}$$

Working in this frame leads us to introduce the oscillator-driving detuning frequency Δ, as defined by

$$\Delta = \omega_0 - \omega_{\mathrm{D}}. \tag{S5}$$

We henceforth consider the detunings $\Delta \geq 0$ without loss of generality, both here and in the main text, since it is the absolute value of this energy difference which is important, rather than its sign. This fact can be seen by looking at the key dynamical matrices $\mathcal{H}$ and $\mathcal{M}$ [cf. Eq. (6) and Eq. (12) in the main text], which are not meaningfully changed after the replacement $\Delta \to -\Delta$ is made within them.

The Hamiltonian $\hat{H}$ of Eq. (S4) is diagonalized with a bosonic Bogoliubov operator β [cf. Eq. (2) from the main text], leading to the following eigenvalue equation for the energy eigenstates $|n, \phi\rangle$ (where the non-negative integer $n = 0, 1, 2, ...$)

$$\hat{H} |n, \phi\rangle = E_n |n, \phi\rangle, \tag{S6}$$

$$E_n = n\tilde{\omega}, \qquad \tilde{\omega} = \sqrt{\Delta^2 - \Omega^2}, \tag{S7}$$

where the necessary condition $\Delta > \Omega$ ensures real eigenvalues E_n. The Bogoliubov eigenstates $|n, \phi\rangle$ are just the squeezed number states $|n, \phi\rangle = S_\phi |n\rangle$, while the number states are denoted by $|n\rangle$. The squeezed ground state satisfies $\beta|0, \phi\rangle = 0$. The squeezing operator S_ϕ, which satisfies the twin relations $S_\phi b S_\phi^\dagger = \beta$ and $S_\phi b^\dagger S_\phi^\dagger = \beta^\dagger$, is defined by

$$S_\phi = \exp\left(\frac{1}{2}\phi \mathrm{e}^{-\mathrm{i}\theta} bb - \frac{1}{2}\phi \mathrm{e}^{\mathrm{i}\theta} b^\dagger b^\dagger\right), \qquad S_\phi^\dagger = S_{-\phi}. \tag{S8}$$

The squeezing parameter ϕ (which enters both β and S_ϕ) is given by the hyperbolic relation $\tanh(2\phi) = \Omega/\Delta$, or equivently

$$\phi = \frac{1}{2} \operatorname{arctanh}\left(\frac{\Omega}{\Delta}\right), \tag{S9}$$

which has solutions for $\Omega < \Delta$, consistent with the regime of wholly real values of E_n as found in Eq. (S7). The energy levels E_n are plotted in Fig. S1 (b) as a function of the dimensionless parameter Ω/Δ, that is the driving amplitude-to-detuning ratio. The results for larger indices n are associated with increasingly thin orange lines. Clearly, at very small drivings $\Omega \ll \Delta$ the standard quantum harmonic oscillator levels equally spaced by Δ are recovered (the levels with $n = 0, 1, 2, 3$ are visible in the plot). At larger drivings $\Omega \lesssim \Delta$ the inter-level spacing is greatly reduced such that a huge amount of levels n are noticeable for some fixed value of the detuning Δ, which hints at the eventual spectral collapse of the system for drivings where $\Omega \geq \Delta$.

II. QUANTUM MASTER EQUATION

The quantum master equation of the system's density matrix ρ is considered to be in the regular Lindbladian form [cf. Eq. (4) from the main text] [S1, S2]

$$\partial_t \rho = \mathrm{i}[\rho, \hat{H}] + \frac{\gamma}{2}\mathscr{L}[b], \qquad \mathscr{L}[b] = 2b\rho b^\dagger - b^\dagger b \rho - \rho b^\dagger b, \tag{S10}$$

where the Hamiltonian $\hat{H}$ is given by Eq. (S4), and where the frequency $\gamma \geq 0$ is the zero-temperature dissipation rate. This open quantum systems model is represented pictorially in the sketch of Fig. S1 (a), where the two-photon drive (red arrow) and one-photon losses (orange arrows) are highlighted. The matrix elements $\rho_{n,m} = \langle n|\rho|m\rangle$ of the density matrix ρ defined through Eq. (S10) are given by the following recurrence equation

$$\begin{aligned} \partial_t \rho_{n,m} = \quad & \rho_{n,m}\left\{\mathrm{i}\,(m-n)\,\Delta - (n+m)\,\frac{\gamma}{2}\right\} + \rho_{n+1,m+1}\left\{\gamma\sqrt{n+1}\sqrt{m+1}\right\} \\ & + \rho_{n,m+2}\left\{\mathrm{i}\frac{\Omega \mathrm{e}^{\mathrm{i}\theta}}{2}\sqrt{m+1}\sqrt{m+2}\right\} + \rho_{n,m-2}\left\{\mathrm{i}\frac{\Omega \mathrm{e}^{-\mathrm{i}\theta}}{2}\sqrt{m}\sqrt{m-1}\right\} \\ & + \rho_{n+2,m}\left\{-\mathrm{i}\frac{\Omega \mathrm{e}^{-\mathrm{i}\theta}}{2}\sqrt{n+1}\sqrt{n+2}\right\} + \rho_{n-2,m}\left\{-\mathrm{i}\frac{\Omega \mathrm{e}^{\mathrm{i}\theta}}{2}\sqrt{n}\sqrt{n-1}\right\}. \end{aligned} \tag{S11}$$

This equation displays the two-photon effect of the parametric driving by linking matrix elements with the indices n or m with $m \pm 2$ or $n \pm 2$ respectively. The finite matrix implied by truncating Eq. (S11) leads to the matrix equation

$$\partial_t \rho = \mathcal{L}\rho, \tag{S12}$$

where ρ is a column vector containing the matrix elements $\rho_{n,m}$ and $\mathcal{L}$ is the Liouvillian square matrix, as used in the calculations leading to Fig. 6 in the main text.

Purity. The purity $\mathcal{P}$ of a quantum state defined by a density matrix ρ is defined as

$$\mathcal{P} = \mathrm{Tr}\left(\rho^2\right), \tag{S13}$$

which has the bounds $1/N \leq \mathcal{P} \leq 1$, where the upper bound $\mathcal{P} = 1$ corresponds to a pure state and the lower bound $\mathcal{P} = 1/N$ to a maximally mixed state. In Fig. S2 (a, b, c) we plot the purity in the steady state $\lim_{t\to\infty} \mathcal{P}$ as a function of the driving amplitude Ω, for a fixed value of the detuning Δ in each panel. We consider increasingly large truncations of the matrix Liouvillian $\mathcal{L}$ (to some large but still finite sizes $N \times N$ matrix), leading to convergences at the points at which the purity $\mathcal{P}$ dramatically

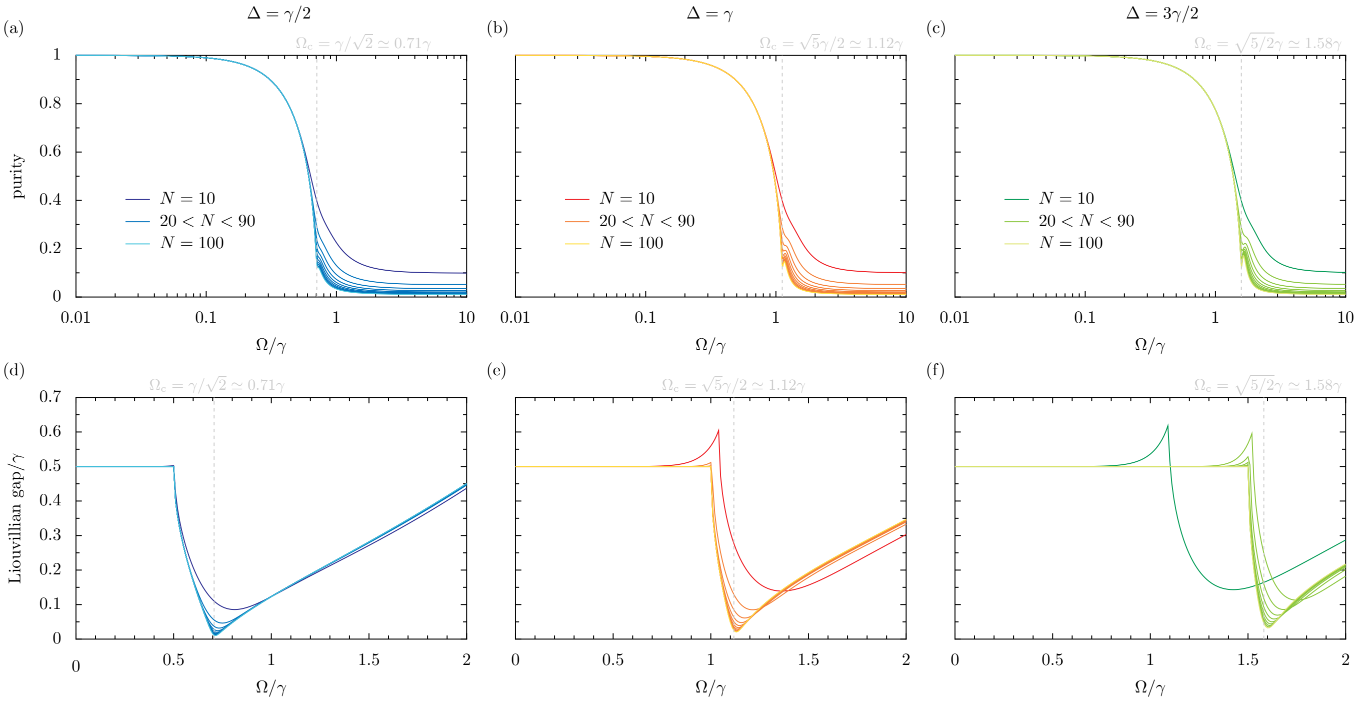

FIG. S2. **Purity and Liouvillian gap of the parametric driven-dissipative oscillator.** Top row: purity in the steady state $\lim_{t\to\infty} \mathcal{P}$ as a function of the driving amplitude Ω, given in units of the damping rate γ. Bottom row: the Liouvillian gap, in units of γ, as a function of Ω. In these calculations, we truncate the matrix Liouvillian $\mathcal{L}$ at some finite size N, where the lowest truncation $N = 10$ (dark lines) and highest truncation $N = 100$ (light lines) are shown, as well as intermediate truncations in steps of 10. Vertical dashed grey lines: the critical driving amplitude $\Omega_c = \sqrt{\Delta^2 + \gamma^2/4}$ [cf. Eq. (16) from the main text]. We consider increasing large detunings Δ across the row of panels. Panels (a, d): $\Delta = \gamma/2$. Panels (b, e) $\Delta = \gamma$. Panel (c, f) $\Delta = 3\gamma/2$.

drops to the lower bound $\mathcal{P} \simeq 1/N$. In the limit thermodynamic limit of $N \to \infty$, such a diagram is reminiscent of a phase transition. Notably, the critical points tend towards the critical driving amplitude $\Omega_c = \sqrt{\Delta^2 + \gamma^2/4}$ [cf. Eq. (16) from the main text], which are marked with dashed lines in each panel in Fig. S2 (a, b, c).

Liouvillian gap. The Liouvillian eigenvalues of the Liouvillian matrix $\mathcal{L}$, for some finite truncation N of the otherwise infinite matrix equation defined by Eq. (S12), can be readily found numerically. The steady state is associated with the zero eigenvalue, and otherwise the closest eigenvalue to 0 (in real value) defines the Liouvillian gap of the system. In Fig. S2 (d, e, f) we plot the Liouvillian gap as a function of the driving amplitude Ω, for a fixed value of the detuning Δ in each panel [cf. panels (a, b, c)]. We consider increasingly large truncations of the matrix Liouvillian $\mathcal{L}$ in the same manner as panels (a, b, c), leading to convergences at the points at which the Liouvillian gap closes. Notably, the Liouvillian gap is seen to close at the critical driving amplitude Ω_c [cf. Eq. (16) from the main text], which are marked with dashed lines in each panel in Fig. S2 (d, e, f). Such closings of the Liouvillian gap are typically associated with a (first-order) dissipative phase transition.

Quadratures. The generalized quadrature operators $\hat{X}$ and $\hat{P}$, which satisfy the commutation relation $[\hat{X}, \hat{P}] = \mathrm{i}$, may be defined as

$$\hat{X} = \frac{1}{\sqrt{2}} \left(\mathrm{e}^{\frac{\mathrm{i}\theta}{2}} b^\dagger + \mathrm{e}^{-\frac{\mathrm{i}\theta}{2}} b \right), \qquad \hat{P} = \frac{\mathrm{i}}{\sqrt{2}} \left(\mathrm{e}^{\frac{\mathrm{i}\theta}{2}} b^\dagger - \mathrm{e}^{-\frac{\mathrm{i}\theta}{2}} b \right), \tag{S14}$$

where the phase θ has entered the definition to counteract its appearance in the Hamiltonian of Eq. (S4). The mean values of $\hat{X}$ and $\hat{P}$ can be found by exploiting the property $\mathrm{Tr}\,(\mathcal{O}\rho) = \langle \mathcal{O} \rangle$, which allows one to access the mean value $\langle \mathcal{O} \rangle$ of any operator $\mathcal{O}$. Using the quantum master equation given as Eq. (S10), the relevant equation of motion is found to be

$$\partial_t \begin{pmatrix} \langle \hat{X} \rangle \\ \langle \hat{P} \rangle \end{pmatrix} = \begin{pmatrix} -\frac{\gamma}{2} & \Delta - \Omega \\ -\Delta - \Omega & -\frac{\gamma}{2} \end{pmatrix} \begin{pmatrix} \langle \hat{X} \rangle \\ \langle \hat{P} \rangle \end{pmatrix}. \tag{S15}$$

In terms of the frequency $\tilde{\omega}$ [as defined in Eq. (S7)], the two complex eigenvalues $\lambda_\pm$ of the above dynamical matrix are

$$\lambda_\pm = -\frac{\gamma}{2} \pm \tilde{\omega}, \tag{S16}$$

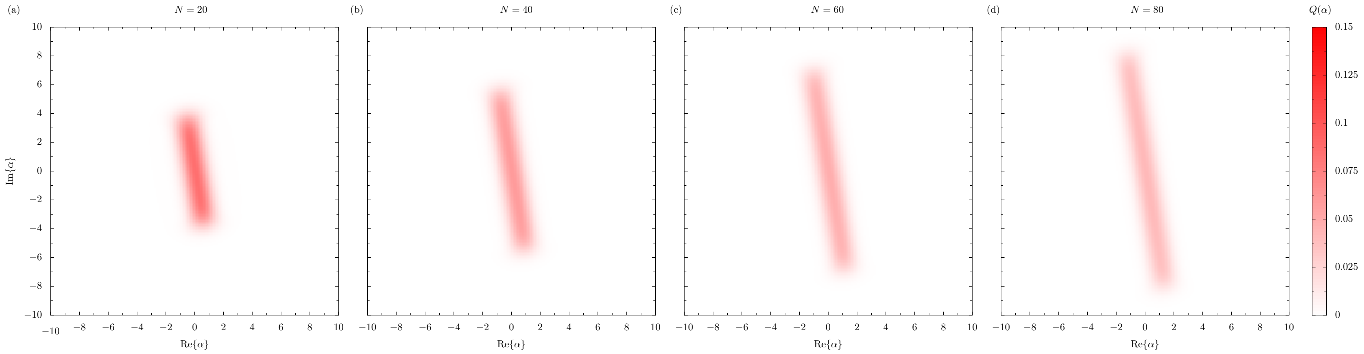

FIG. S3. **The phase-space quasiprobability distribution of the parametric driven-dissipative oscillator at critical driving.** The Husimi function $Q(\alpha) = \langle \alpha | \hat{\rho} | \alpha \rangle / \pi$, or the expectation value of the density operator $\hat{\rho}$ with respect to the coherent state $|\alpha\rangle$, at the critical driving amplitude $\Omega = \Omega_{\mathrm{c}}$. A truncated oscillator with an increasing number of levels N is considered across the row of panels. Panel (a): $N = 20$. Panel (b): $N = 40$. Panel (c): $N = 60$. Panel (d): $N = 80$. In this figure, we consider the case of the detuning $\Delta = 3\gamma/2$, where the damping rate is γ. The Q-function is calculated in the steady state ($t \to \infty$).

and the solution of Eq. (S15), subject to the initial conditions of $\langle \hat{X} \rangle = 0$ and $\langle \hat{P} \rangle = 1$ at some initial time $t = 0$, reads

$$\langle \hat{X} \rangle = \frac{\Delta - \Omega}{\tilde{\omega}} \sin(\tilde{\omega} t) \, \mathrm{e}^{-\frac{\gamma}{2} t}, \qquad \langle \hat{P} \rangle = \cos(\tilde{\omega} t) \, \mathrm{e}^{-\frac{\gamma}{2} t}. \tag{S17}$$

Importantly, the twin eigenvalues of Eq. (S16) suggest a point of importance at the degeneracy $\lambda_+ = \lambda_-$, where $\Omega = \Delta$. In the main text, this is revealed to be an exceptional point of the system. The main text also considers the squared quantities $\hat{X}^2$ and $\hat{P}^2$, defined via Eq. (S14) as

$$\hat{X}^2 = \frac{1}{2} \left(1 + 2b^\dagger b + \mathrm{e}^{\mathrm{i}\theta} b^\dagger b^\dagger + \mathrm{e}^{-\mathrm{i}\theta} bb \right), \qquad \hat{P}^2 = \frac{1}{2} \left(1 + 2b^\dagger b - \mathrm{e}^{\mathrm{i}\theta} b^\dagger b^\dagger - \mathrm{e}^{-\mathrm{i}\theta} bb \right), \tag{S18}$$

which enter, in their averaged form, in the quadrature variances $\sigma^2_{X,P}$ discussed around Eq. (29) and Fig. 5 (e) in the main text. In the steady state, the quantities necessary to arrive at Eq. (29) are $\lim_{t\to\infty} \langle X \rangle = \lim_{t\to\infty} \langle P \rangle = 0$, as well as $\lim_{t\to\infty} \langle X^2 \rangle$ and $\lim_{t\to\infty} \langle P^2 \rangle$, which may be determined from Eq. (S18) with the steady state solutions of the second moments equation [cf. Eq. (12) in the main text].

Phase-space quasiprobability distribution. In the main text, the Husimi function $Q(\alpha) = \langle \alpha | \hat{\rho} | \alpha \rangle / \pi$ was considered as a measure of the phase-space quasiprobability distribution of the parametric driven-dissipative oscillator, and the steady state ($t \to \infty$) was examined in particular. In the thermodynamic limit of an untruncated oscillator, where the number of levels $N \to \infty$, the steady state population exhibits an interesting point at the driving amplitude $\Omega = \Omega_{\mathrm{c}}$. In Fig. 5 (a–d) of the main text, we showed the evolution of the steady state Q-function with increasing driving amplitude Ω, for an oscillator with $N = 40$ levels. Panel (d) of Fig. 5 shows the result at the critical driving $\Omega = \Omega_{\mathrm{c}}$, where the Q-function takes on the unusual appearance of a diluted cigar. In Fig. S3 we hold the driving amplitude constant at Ω_{c} and instead increase the number of levels N across the row of panels. The figure shows that with an increasingly large number of levels the Q-function becomes increasingly long, narrow and diluted, such that in the untruncated limit it disappears ($\lim_{N\to\infty} Q(\alpha) \to 0$). This should be the case due to the absence of a steady state for a proper quantum harmonic oscillator in this parameter regime.

III. FIRST-ORDER COHERENCE

The degree of first-order coherence $g^{(1)}(\tau)$, after some time delay τ and normalized by the steady state population $\langle b^\dagger b \rangle$ of the oscillator, is defined as [S1, S2]

$$g^{(1)}(\tau) = \lim_{t\to\infty} \frac{\langle b^\dagger(t) b(t+\tau) \rangle}{\langle b^\dagger(t) b(t) \rangle}. \tag{S19}$$

The quantum regression formula and the quantum master equation of Eq. (S10) together suggest the desired equation for the mean value of the two-time operator $b^\dagger(t) b(t+\tau)$ [cf. Eq. (5) from the main text]

$$\partial_\tau \mathbf{v} + \mathbf{H} \mathbf{v} = 0, \tag{S20}$$

where the column vector $\mathbf{v}$, which collects the correlators, and the dynamical matrix $\mathbf{H}$ are given by

$$\mathbf{v}(t,t+\tau) = \begin{pmatrix} \langle b^\dagger(t) b(t+\tau) \rangle \\ \langle b^\dagger(t) b^\dagger(t+\tau) \rangle \end{pmatrix}, \qquad \mathbf{H} = \begin{pmatrix} \mathrm{i}\Delta + \frac{\gamma}{2} & \mathrm{i}\Omega \mathrm{e}^{\mathrm{i}\theta} \\ -\mathrm{i}\Omega \mathrm{e}^{-\mathrm{i}\theta} & -\mathrm{i}\Delta + \frac{\gamma}{2} \end{pmatrix}. \tag{S21}$$

The degree of first-order coherence $g^{(1)}(\tau)$ is discussed around Eq. (20)–(22) in the main text.

IV. SECOND-ORDER COHERENCE

The degree of second-order coherence $g^{(2)}(\tau)$, after a time delay τ and normalized by the steady state result found at long times scales, is given by [S1, S2]

$$g^{(2)}(\tau) = \lim_{t\to\infty} \frac{\langle b^\dagger(t) b^\dagger(t+\tau) b(t+\tau) b(t) \rangle}{\langle b^\dagger(t) b(t) \rangle^2}. \tag{S22}$$

The quantum master equation of Eq. (S10) yields the following equation of motion for the fourth moments [cf. Eq. (11) from the main text]

$$\partial_\tau \mathbf{u} = \mathbf{P} \langle b^\dagger(t) b(t) \rangle - \mathbf{M}\mathbf{u}, \tag{S23}$$

where the column vector $\mathbf{u}$ of two-time correlators, the drive term $\mathbf{P}$, and the dynamical matrix $\mathbf{M}$ are given by

$$\mathbf{u} = \begin{pmatrix} \langle b^\dagger(t) b^\dagger(t+\tau) b(t+\tau) b(t) \rangle \\ \langle b^\dagger(t) b(t+\tau) b(t+\tau) b(t) \rangle \\ \langle b^\dagger(t) b^\dagger(t+\tau) b^\dagger(t+\tau) b(t) \rangle \end{pmatrix}, \quad \mathbf{P} = \begin{pmatrix} 0 \\ -\mathrm{i}\Omega \mathrm{e}^{\mathrm{i}\theta} \\ \mathrm{i}\Omega \mathrm{e}^{-\mathrm{i}\theta} \end{pmatrix}, \quad \mathbf{M} = \begin{pmatrix} \gamma & -\mathrm{i}\Omega \mathrm{e}^{-\mathrm{i}\theta} & \mathrm{i}\Omega \mathrm{e}^{\mathrm{i}\theta} \\ 2\mathrm{i}\Omega \mathrm{e}^{\mathrm{i}\theta} & 2\mathrm{i}\Delta + \gamma & 0 \\ -2\mathrm{i}\Omega \mathrm{e}^{-\mathrm{i}\theta} & 0 & -2\mathrm{i}\Delta + \gamma \end{pmatrix}. \tag{S24}$$

The solution of Eq. (S23), together with the definition of Eq. (S22), leads to the expressions for $g^{(2)}(\tau)$ discussed around Eq. (23)–(26) in the main text.

V. OPTICAL SPECTRUM

The optical spectrum $S(\omega)$ may be defined through the integral of the two-time correlator $\langle b^\dagger(t) b(t+\tau) \rangle$, which is in turn found by solving Eq. (S20), as follows [S1, S2]

$$S(\omega) = \lim_{t\to\infty} \frac{1}{\pi} \frac{\mathrm{Re} \int_0^\infty \langle b^\dagger(t) b(t+\tau) \rangle \mathrm{e}^{\mathrm{i}\omega\tau} \mathrm{d}\tau}{\langle b^\dagger(t) b(t) \rangle}, \tag{S25}$$

which has been normalized using the steady state of the mean population, so that $\int_\infty^\infty S(\omega)\mathrm{d}\omega = 1$. Since $\langle b^\dagger(t) b(t+\tau) \rangle$ is in general composed of two terms [cf. Eq. (S21)], both of which being products of an exponential in τ and a complex number, the integral in Eq. (S25) can be analytically performed, leading to the standard result

$$S(\omega) = \sum_{p=+,-} S_p, \tag{S26}$$

where the spectral lineshape S_p, corresponding to the contribution of each of the two terms, can de decomposed into the general forms

$$S_p(\omega) = \frac{L_p}{\pi} \frac{\frac{\gamma_p}{2}}{\left(\frac{\gamma_p}{2}\right)^2 + (\omega - \omega_p)^2} - \frac{K_p}{\pi} \frac{(\omega - \omega_p)}{\left(\frac{\gamma_p}{2}\right)^2 + (\omega - \omega_p)^2}, \tag{S27}$$

where L_p and K_p are the real-valued weighting coefficients of the Lorentzian and dispersive parts respectively, while ω_p and γ_p define the positions of the spectral resonances and their spectral linewidths. The later two quantities arise as the real and imaginary parts of the complex eigenvalues of the matrix $\mathbf{H}$, which is given in Eq. (S21). As discussed in the main text [cf. Fig. 4], the optical spectrum may present either a doublet or a singlet depending upon the driving amplitude Ω.

A. Doublet regime

In this case of weaker drivings $\Omega < \Delta$, then the complex eigenvalues of $\mathbf{H}$ [cf. Eq. (S21)] suggest the following complex eigenvalue ϵ_p decomposition into imaginary and real parts, $\mathrm{Re}\{\epsilon_p\} = \gamma_p/2$ and $\mathrm{Im}\{\epsilon_p\} = \omega_p$, as follows

$$\gamma_+ = \gamma, \qquad \omega_+ = +\tilde{\omega}, \tag{S28a}$$
$$\gamma_- = \gamma, \qquad \omega_- = -\tilde{\omega}, \tag{S28b}$$

where $\tilde{\omega} = \sqrt{\Delta^2 - \Omega^2}$. The complex coefficients of the solution of Eq. (S20), coming from the associated eigenvectors of $\mathbf{H}$, enables the amplitude decomposition into L_p and K_p [cf. Eq. (S26)] as

$$L_+ = \frac{1}{2}, \qquad K_+ = +\frac{\gamma}{4\tilde{\omega}}, \tag{S29a}$$
$$L_- = \frac{1}{2}, \qquad K_- = -\frac{\gamma}{4\tilde{\omega}}. \tag{S29b}$$

Substitution of the broadenings and resonances of Eq. (S28), along with the weightings of Eq. (S29), into the spectrum definition of Eq. (S26) and Eq. (S27) finally leads to the doublet spectrum

$$S(\omega) = \sum_{p=+,-} \left\{ \frac{1}{2\pi} \frac{\frac{\gamma}{2}}{\left(\frac{\gamma}{2}\right)^2 + (\omega + p\tilde{\omega})^2} + \frac{p\gamma}{4\tilde{\omega}\pi} \frac{(\omega + p\tilde{\omega})}{\left(\frac{\gamma}{2}\right)^2 + (\omega + p\tilde{\omega})^2} \right\}, \qquad (\Omega \leq \Delta)\,, \tag{S30}$$

which has been rewritten as Eq. (27) in the main text. The two contributions S_+ and S_- are plotted as the thin cyan and orange lines in Fig. 4 in the main text.

B. Singlet regime

The analysis of the singlet regime proceeds in a similar manner. For stronger drivings $\Omega > \Delta$, the complex eigenvalues ϵ_p may instead be decomposed as [cf. Eq. (S28)]

$$\gamma_+ = \gamma - 2\Gamma, \qquad \omega_+ = 0, \tag{S31a}$$
$$\gamma_- = \gamma + 2\Gamma, \qquad \omega_- = 0, \tag{S31b}$$

such that there is notably no shift in resonance frequency, since $\omega_p = 0$. The weighting coefficients follow as [cf. Eq. (S29)]

$$L_+ = \frac{1}{2} + \frac{\gamma}{4\Gamma}, \qquad K_+ = 0, \tag{S32a}$$
$$L_- = \frac{1}{2} - \frac{\gamma}{4\Gamma}, \qquad K_- = 0, \tag{S32b}$$

which present wholly Lorentzian contributions since $K_p = 0$. The overall singlet spectrum $S(\omega)$ therefore is [cf. Eq. (S30)]

$$S(\omega) = \sum_{p=+,-} \frac{\frac{1}{2} + \frac{p\gamma}{4\Gamma}}{\pi} \frac{\frac{\gamma - 2p\Gamma}{2}}{\left(\frac{\gamma - 2p\Gamma}{2}\right)^2 + \omega^2}, \qquad (\Omega \geq \Delta)\,, \tag{S33}$$

as written as Eq. (27) in the main text.

VI. SECOND AND FOURTH MOMENTS

The quantum master equation of Eq. (S10) yields the following equation of motion for the second and fourth moments of the parametrically driven oscillator

$$\mathrm{i}\partial_t \mathbf{w} = \mathbf{L}\mathbf{w} + \mathbf{Q}, \tag{S34}$$

for the 8-vector of correlators $\mathbf{w}$ and the drive term $\mathbf{Q}$, as defined by

$$\mathbf{w} = \begin{pmatrix} \langle b^\dagger b\rangle \\ \langle bb\rangle \\ \langle b^\dagger b^\dagger\rangle \\ \langle b^\dagger b^\dagger bb\rangle \\ \langle bbbb\rangle \\ \langle b^\dagger b^\dagger b^\dagger b^\dagger\rangle \\ \langle b^\dagger bbb\rangle \\ \langle b^\dagger b^\dagger b^\dagger b\rangle \end{pmatrix}, \qquad \mathbf{Q} = \begin{pmatrix} 0 \\ \Omega \mathrm{e}^{\mathrm{i}\theta} \\ -\Omega \mathrm{e}^{-\mathrm{i}\theta} \\ 0 \\ 0 \\ 0 \\ 0 \\ 0 \end{pmatrix}, \tag{S35}$$

and where the dynamical matrix $\mathbf{L}$ reads

$$\mathbf{L} = \begin{pmatrix} -\mathrm{i}\gamma & -\Omega \mathrm{e}^{-\mathrm{i}\theta} & \Omega \mathrm{e}^{\mathrm{i}\theta} & 0 & 0 & 0 & 0 & 0 \\ 2\Omega \mathrm{e}^{\mathrm{i}\theta} & 2\Delta - \mathrm{i}\gamma & 0 & 0 & 0 & 0 & 0 & 0 \\ -2\Omega \mathrm{e}^{-\mathrm{i}\theta} & 0 & -2\Delta - \mathrm{i}\gamma & 0 & 0 & 0 & 0 & 0 \\ 0 & -\Omega \mathrm{e}^{-\mathrm{i}\theta} & \Omega \mathrm{e}^{\mathrm{i}\theta} & -2\mathrm{i}\gamma & 0 & 0 & -2\Omega \mathrm{e}^{-\mathrm{i}\theta} & 2\Omega \mathrm{e}^{\mathrm{i}\theta} \\ 0 & 6\Omega \mathrm{e}^{\mathrm{i}\theta} & 0 & 0 & 4\Delta - 2\mathrm{i}\gamma & 0 & 4\Omega \mathrm{e}^{\mathrm{i}\theta} & 0 \\ 0 & 0 & -6\Omega \mathrm{e}^{-\mathrm{i}\theta} & 0 & 0 & -4\Delta - 2\mathrm{i}\gamma & 0 & -4\Omega \mathrm{e}^{-\mathrm{i}\theta} \\ 3\Omega \mathrm{e}^{\mathrm{i}\theta} & 0 & 0 & 3\Omega \mathrm{e}^{\mathrm{i}\theta} & -\Omega \mathrm{e}^{-\mathrm{i}\theta} & 0 & 2\Delta - 2\mathrm{i}\gamma & 0 \\ -3\Omega \mathrm{e}^{-\mathrm{i}\theta} & 0 & 0 & -3\Omega \mathrm{e}^{-\mathrm{i}\theta} & 0 & \Omega \mathrm{e}^{\mathrm{i}\theta} & 0 & -2\Delta - 2\mathrm{i}\gamma \end{pmatrix}. \tag{S36}$$

In the steady state, one obtains from Eq. (S34) the following analytic expressions for the eight mean correlators

$$\lim_{t\to\infty} \langle b^\dagger b\rangle = \frac{1}{2} \frac{\Omega^2}{\Delta^2 + \left(\frac{\gamma}{2}\right)^2 - \Omega^2}, \tag{S37}$$

$$\lim_{t\to\infty} \langle bb\rangle = -\frac{\Omega \mathrm{e}^{\mathrm{i}\theta}}{2} \frac{\Delta + \mathrm{i}\frac{\gamma}{2}}{\Delta^2 + \left(\frac{\gamma}{2}\right)^2 - \Omega^2}, \qquad \lim_{t\to\infty} \langle b^\dagger b^\dagger\rangle = \lim_{t\to\infty} \langle bb\rangle^*, \tag{S38}$$

$$\lim_{t\to\infty} \langle b^\dagger b^\dagger bb\rangle = \frac{\Omega^2}{4} \frac{\Delta^2 + \left(\frac{\gamma}{2}\right)^2 + 2\Omega^2}{\left(\Delta^2 + \left(\frac{\gamma}{2}\right)^2 - \Omega^2\right)^2}, \tag{S39}$$

$$\lim_{t\to\infty} \langle bbbb\rangle = \frac{3\Omega^2 \mathrm{e}^{2\mathrm{i}\theta}}{4} \frac{\left(\Delta + \mathrm{i}\frac{\gamma}{2}\right)^2}{\left(\Delta^2 + \left(\frac{\gamma}{2}\right)^2 - \Omega^2\right)^2}, \qquad \lim_{t\to\infty} \langle b^\dagger b^\dagger b^\dagger b^\dagger\rangle = \lim_{t\to\infty} \langle bbbb\rangle^*, \tag{S40}$$

$$\lim_{t\to\infty} \langle b^\dagger bbb\rangle = \frac{-3\Omega^3 \mathrm{e}^{\mathrm{i}\theta}}{4} \frac{\Delta + \mathrm{i}\frac{\gamma}{2}}{\left(\Delta^2 + \left(\frac{\gamma}{2}\right)^2 - \Omega^2\right)^2}, \qquad \lim_{t\to\infty} \langle b^\dagger b^\dagger b^\dagger b\rangle = \lim_{t\to\infty} \langle b^\dagger bbb\rangle^*. \tag{S41}$$

The above results, along with the definition of Eq. (S22), lead to the expression for the second-order coherence at zero time delay

$$g^{(2)}(0) = \lim_{t\to\infty} \frac{\langle b^\dagger(t) b^\dagger(t) b(t) b(t)\rangle}{\langle b^\dagger(t) b(t)\rangle^2} = 2 + \frac{\Delta^2 + \left(\frac{\gamma}{2}\right)^2}{\Omega^2}, \tag{S42}$$

as further discussed in the main text around Eq. (25). Notably, this zero temperature result is dependent on the order one takes limits (see the nonzero temperature analysis provided later on).

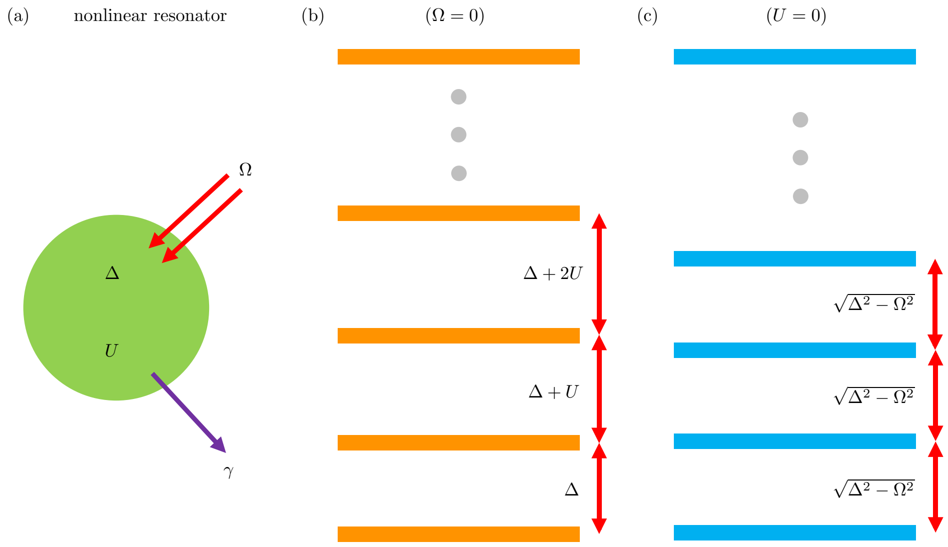

FIG. S4. **The parametric driven-dissipative nonlinear oscillator.** Panel (a): a sketch of the nonlinear oscillator (green circle), including the detuning Δ and the interaction strength U [cf. Eq. (S45)]. The parametric driving amplitude is Ω (red arrows), and the damping decay rate is γ (purple arrow). Panel (b): The infinite and bosonic energy ladder of the system in the undriven limit ($\Omega \to 0$) [cf. Eq. (S46)]. Panel (c): The energy ladder of the system in the linear limit ($U \to 0$) [cf. Eq. (S47)].

VII. INTERACTIONS

The Hamiltonian of Eq. (S1) may be supplemented by an interaction, Kerr-like term to describe an anharmonic oscillator. Such a nonlinear Hamiltonian $\hat{\mathcal{H}}$ is then given by

$$\hat{\mathcal{H}} = \hat{\mathcal{H}}_0 + \hat{\mathcal{H}}_{\mathrm{I}} + \hat{\mathcal{H}}_{\mathrm{D}}, \tag{S43}$$

where the interactions term $\hat{\mathcal{H}}_{\mathrm{I}}$, where $U \geq 0$ measures the strength of the repulsive nonlinearity, is defined by

$$\hat{\mathcal{H}}_{\mathrm{I}} = \frac{U}{2} b^\dagger b^\dagger b b. \tag{S44}$$

A transformation of the form of Eq. (S3) leads to the rotated Hamiltonian $\hat{H}$ [cf. Eq. (S4) for the linear version]

$$\hat{H} = \Delta b^\dagger b + \frac{U}{2} b^\dagger b^\dagger b b + \frac{\Omega \mathrm{e}^{\mathrm{i}\theta}}{2} b^\dagger b^\dagger + \frac{\Omega \mathrm{e}^{-\mathrm{i}\theta}}{2} b b, \tag{S45}$$

as is represented in the sketch in Fig. S4 (a).

In two limiting cases, the eigenenergies E_n associated with Eq. (S45) are particularly simple. Let us consider (i) when the parametric driving is vanishing ($\Omega \to 0$), and (ii) when the anharmonicity is negligible ($U \to 0$). In these cases, the following analytic expressions for the eigenenergies E_n and energetic separations $E_{n+1} - E_n$ arise

$$E_n = n\Delta + \frac{n\,(n-1)}{2} U, \qquad E_{n+1} - E_n = \Delta + nU, \qquad (\Omega \to 0) \tag{S46}$$

$$E_n = n\sqrt{\Delta^2 - \Omega^2}, \qquad E_{n+1} - E_n = \sqrt{\Delta^2 - \Omega^2}, \qquad (U \to 0). \tag{S47}$$

The undriven case of Eq. (S46) exhibits the additional energy cost nU of transitions between successive levels due to the anharmonic term, as is drawn as the orange energy ladder in Fig. S4 (b). The harmonic limit of Eq. (S47) shows n-independent transitions as displayed in the cyan energy ladder of Fig. S4 (c), and in this regime one again requires sufficient detunings $\Delta > \Omega$ in order to have real eigenenergies E_n.

The full and exact energies $E_n = E_n(\Delta, \Omega, U)$ for the intermediate cases between these two aforementioned extremes – $E_n = E_n(\Delta, 0, U)$ and $E_n = E_n(\Delta, \Omega, 0)$ as provided in Eq. (S46) and Eq. (S47) respectively – can be computed from the Hamiltonian matrix elements $\langle m|\hat{H}|n\rangle$, which are given by

$$\langle m|\hat{H}|n\rangle = \left\{ n\Delta + \frac{n(n-1)}{2} U \right\} \langle m|n\rangle + \left\{ \sqrt{n+1}\sqrt{n+2}\, \frac{\Omega \mathrm{e}^{\mathrm{i}\theta}}{2} \right\} \langle m|n+2\rangle + \left\{ \sqrt{n}\sqrt{n-1}\, \frac{\Omega \mathrm{e}^{-\mathrm{i}\theta}}{2} \right\} \langle m|n-2\rangle, \tag{S48}$$

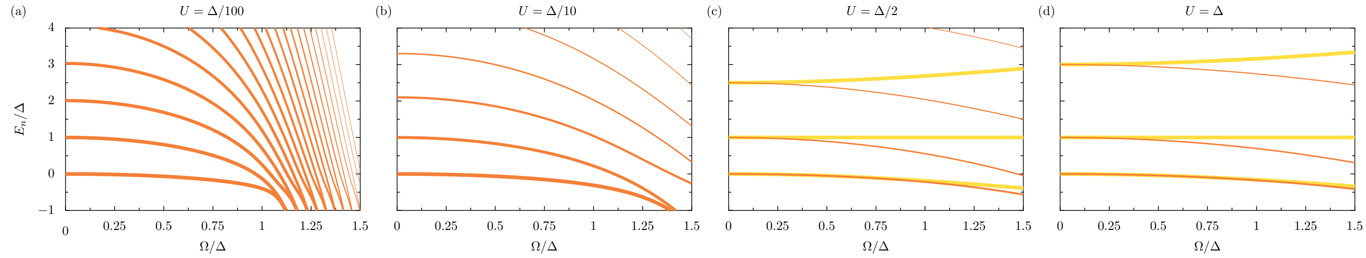

FIG. S5. **Energy levels of the parametric driven-dissipative nonlinear oscillator.** The lowest eigenenergies E_n (where $n = 0, 1, ..., 20$) as a function of the driving amplitude Ω. Panel (a): The interaction strength $U = \Delta/100$. Panel (b): $U = \Delta/10$. Panel (c): $U = \Delta/2$. Panel (d): $U = \Delta$. All quantities are given in units of the detuning Δ. The eigenenergies were found by diagonalizing the infinite matrix given by Eq. (S48), which was truncated with the dimensions 150×150. Thick yellow lines in panels (c) and (d): analytic results for the three lowest levels after a 3×3 truncation [Eq. (S50)].

which helps to define an infinitely large square matrix, which in practice can be truncated at some reasonably large size $N \times N$, creating a square Hamiltonian matrix H_N. For example, in Fig. S5 we show the energy levels of the parametric driven-dissipative nonlinear oscillator as a function of the driving amplitude Ω, for increasing values of the interaction strength U across the row of columns and for a system size of $N = 150$. In all panels, the vanishing driving result of Eq. (S46) may be observed towards the vertical axis. In panel (a), where the anharmonicity is small ($U = \Delta/100$), the result resembles the harmonic results plotted in Fig. S1 (b) and described with Eq. (S47), except for the blurring of the convergence point of all energies away from $\Omega = \Delta$. For larger anharmonicities U, especially in panels (c) and (d), drastically smaller truncations give a reasonable approximation to the lowest energies (the thick yellow lines in these panels are given by analytic expressions after a somewhat brutal truncation at $N = 3$).

Three-level truncation. The case of truncation at just three levels ($N = 3$) suggests that Eq. (S48) reduces to a 3×3 Hamiltonian H_3, which may be represented as

$$H_3 = \begin{pmatrix} 0 & 0 & \frac{\Omega e^{i\theta}}{\sqrt{2}} \\ 0 & \Delta & 0 \\ \frac{\Omega e^{-i\theta}}{\sqrt{2}} & 0 & 2\Delta + U \end{pmatrix}, \tag{S49}$$

whose three eigenvalues E_0, E_1 and E_2 respectively are given by

$$E_0 = \Delta + \frac{U}{2} - \sqrt{\frac{\Omega^2}{2} + \left(\Delta + \frac{U}{2}\right)^2}, \qquad E_1 = \Delta, \qquad E_2 = \Delta + \frac{U}{2} + \sqrt{\frac{\Omega^2}{2} + \left(\Delta + \frac{U}{2}\right)^2}. \tag{S50}$$

The simple expressions of Eq. (S50) are plotted as the thick yellow lines in panels (c) and (d) of Fig. S5, which match relatively well with the numerically generated lower energies E_n (thin orange lines) for not too large ratios of Ω/Δ. Importantly, this result demonstrates analytically how the issue of complex Hamiltonian eigenvalues E_n can be avoided by truncating the infinite system.

A. Liouvillian

The anharmonic oscillator Hamiltonian $\hat{H}$ of Eq. (S45), along with the quantum master equation of Eq. (S10), leads to the following first-order differential equation governing the dynamics of a density matrix element $\rho_{n,m}$ [cf. Eq. (S11) for the purely harmonic case]

$$\begin{aligned} \partial_t \rho_{n,m} = \quad & \rho_{n,m} \left\{ i \left(m - n\right) \Delta - \left(n + m\right) \frac{\gamma}{2} + i \Big[m \left(m - 1\right) - n \left(n - 1\right) \Big] \frac{U}{2} \right\} \\ & + \rho_{n+1,m+1} \left\{ \gamma \sqrt{n+1} \sqrt{m+1} \right\} \\ & + \rho_{n,m+2} \left\{ i \frac{\Omega e^{i\theta}}{2} \sqrt{m+1} \sqrt{m+2} \right\} + \rho_{n,m-2} \left\{ i \frac{\Omega e^{-i\theta}}{2} \sqrt{m} \sqrt{m-1} \right\} \\ & + \rho_{n+2,m} \left\{ - i \frac{\Omega e^{-i\theta}}{2} \sqrt{n+1} \sqrt{n+2} \right\} + \rho_{n-2,m} \left\{ - i \frac{\Omega e^{i\theta}}{2} \sqrt{n} \sqrt{n-1} \right\}. \end{aligned} \tag{S51}$$

The above equation suggests the matrix form $\partial_t \rho = \mathcal{L}\rho$, where the square matrix Liouvillian $\mathcal{L}$ allows one to calculate, for example, the Liouvillian eigenvalues and Liouvillian gaps (which are considered in detail in Fig. 6 in the main text for various truncated cases).

B. Correlators

In general, the equations needed to calculate the mean value of some correlator made up of a string of operators like ${b^\dagger}^n b^m$, where n and m are non-negative integers, follows from the anharmonic Hamiltonian $\hat{H}$ of Eq. (S45) and the quantum master equation of Eq. (S10) like so

$$
\begin{aligned}
\mathrm{i}\partial_t \left\langle {b^\dagger}^n b^m \right\rangle = \; & \left\langle {b^\dagger}^n b^m \right\rangle \left\{ (m-n)\,\Delta - \mathrm{i}\left(\frac{n+m}{2}\right)\gamma + \Big[m\,(m-1) - n\,(n-1) \Big] \frac{U}{2} \right\} \\
& + \left\langle {b^\dagger}^{(n+1)} b^{(m-1)} \right\rangle \left\{ m\Omega \mathrm{e}^{\mathrm{i}\theta} \right\} + \left\langle {b^\dagger}^{n} b^{(m-2)} \right\rangle \left\{ m\,(m-1)\,\frac{\Omega \mathrm{e}^{\mathrm{i}\theta}}{2} \right\} \\
& + \left\langle {b^\dagger}^{(n-1)} b^{(m+1)} \right\rangle \left\{ -\, n\Omega \mathrm{e}^{-\mathrm{i}\theta} \right\} + \left\langle {b^\dagger}^{(n-2)} b^{m} \right\rangle \left\{ -\, n\,(n-1)\,\frac{\Omega \mathrm{e}^{-\mathrm{i}\theta}}{2} \right\} \\
& + \left\langle {b^\dagger}^{(n+1)} b^{(m+1)} \right\rangle \left\{ U\,(m-n) \right\}.
\end{aligned}
\tag{S52}
$$

In this anharmonic case of $U \neq 0$ the above equations never close and so one needs to truncate the system at some reasonably large size depending upon the system parameters considered. However, the simplest nontrivial case of truncation provides some insight into more complicated cases.

Odd truncation at the third-moments. The case of truncation at the third-moments only leads to the following six-dimensional system of equations

$$
\partial_t \psi_3 = \mathcal{H}_3 \psi_3, \qquad\qquad \psi_3 = \begin{pmatrix} \langle b \rangle \\ \langle b^\dagger \rangle \\ \langle b^\dagger b b \rangle \\ \langle b^\dagger b^\dagger b \rangle \\ \langle b b b \rangle \\ \langle b^\dagger b^\dagger b^\dagger \rangle \end{pmatrix},
\tag{S53}
$$

where ψ_3 collects the first-moments and third-moments, and the effective Hamiltonian matrix $\mathcal{H}_3$ reads

$$
\mathcal{H}_3 = \begin{pmatrix}
\Delta - \mathrm{i}\frac{\gamma}{2} & \Omega \mathrm{e}^{\mathrm{i}\theta} & U & 0 & 0 & 0 \\
-\Omega \mathrm{e}^{-\mathrm{i}\theta} & -\Delta - \mathrm{i}\frac{\gamma}{2} & 0 & -U & 0 & 0 \\
0 & \Omega \mathrm{e}^{\mathrm{i}\theta} & \Delta + U - \mathrm{i}\frac{3\gamma}{2} & 2\Omega \mathrm{e}^{\mathrm{i}\theta} & -\Omega \mathrm{e}^{-\mathrm{i}\theta} & 0 \\
-\Omega \mathrm{e}^{-\mathrm{i}\theta} & 0 & -2\Omega \mathrm{e}^{-\mathrm{i}\theta} & -\Delta - U - \mathrm{i}\frac{3\gamma}{2} & 0 & \Omega \mathrm{e}^{\mathrm{i}\theta} \\
3\Omega \mathrm{e}^{\mathrm{i}\theta} & 0 & 3\Omega \mathrm{e}^{\mathrm{i}\theta} & 0 & 3\Delta + 3U - \mathrm{i}\frac{3\gamma}{2} & 0 \\
0 & -3\Omega \mathrm{e}^{-\mathrm{i}\theta} & 0 & -3\Omega \mathrm{e}^{-\mathrm{i}\theta} & 0 & -3\Delta - 3U - \mathrm{i}\frac{3\gamma}{2}
\end{pmatrix}.
\tag{S54}
$$

The top left-hand side 2×2 sub-matrix of the effective Hamiltonian of Eq. (S54) is familiar as the first-moments matrix for the harmonic system, as is given in Eq. (6) in the main text, and suggests the harmonic limit exceptional point at $\Omega = \Delta$. The nonlinearity U couples all of the odd-valued moments, which due to the truncation applied here leads to the 6×6 matrix $\mathcal{H}_3$. Then the diagonalization of Eq. (S54) allows one to find the multiple points of complex eigenvalue coalescence in this anharmonic case, as is shown in Fig. S6. In this figure, panels (a) and (c) shows the real and imaginary parts of the complex eigenvalues for the harmonic case ($U = 0$) which reproduces the expected coalescence point at $\Omega = \Delta$ as previously found from the linear equations [cf. Eq. (6) in the main text]. In panels (b) and (d) a typical anharmonic case is shown ($U = \gamma/100$), which implies the existence of three distinct coalescence points (marked by the thin, grey vertical lines). In this way, the effect of nonlinearities is seen to lead to an increased number of points of coalescence depending upon the truncation (the choice of which physically depends upon the values of the selected parameters of the problem). Hence, the harmonic treatment of the main text holds for sufficiently small anharmonicities.

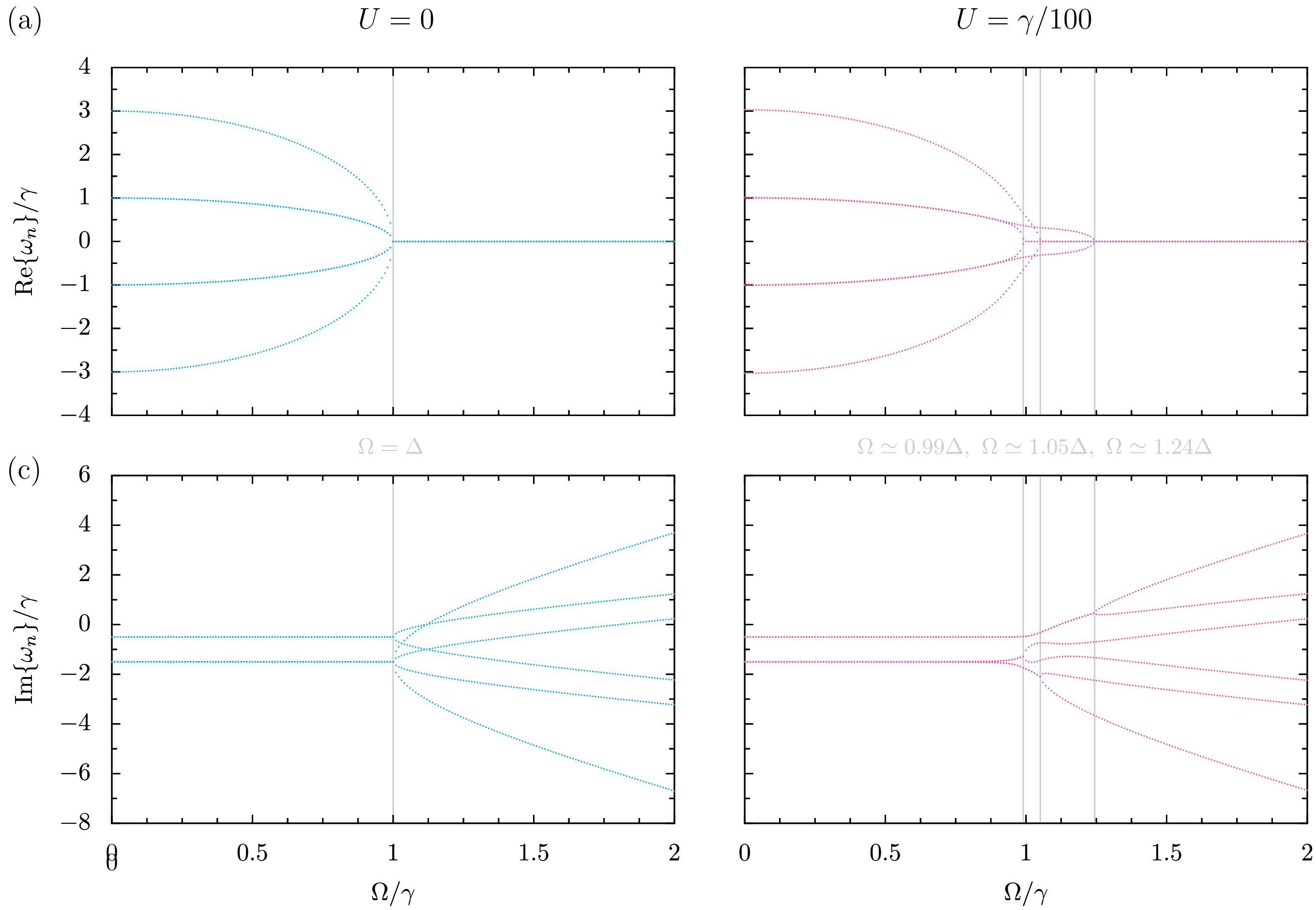

FIG. S6. **Complex eigenfrequencies of the parametric driven-dissipative nonlinear oscillator with truncation.** The six eigenfrequencies ω_n as a function of the driving amplitude Ω, both in units of the loss rate γ, and found from the eigenvalues of Eq. (S54). Upper row: The real parts of ω_n. Lower row: The imaginary parts of ω_n. Left column: the interaction strength $U = 0$. Right column: the interaction strength $U = \gamma/100$. Vertical gray lines: points where ω_n coalesce. In the figure, we consider the detuning $\Delta = \gamma$.

C. Semi-classical approximation

The pair of coupled equations for the first moments $\langle b \rangle$ and $\langle b^\dagger \rangle$, as defined by Eq. (5) in the main text for the harmonic oscillator, need to be upgraded to take account for the nonlinearity U of the anharmonic oscillator. Using Eq. (S52), one finds the two nonlinear equations

$$\partial_t \langle b \rangle = -\left(\mathrm{i}\Delta + \frac{\gamma}{2}\right)\langle b \rangle - \mathrm{i}\Omega \mathrm{e}^{\mathrm{i}\theta}\langle b^\dagger \rangle - \mathrm{i}U\langle b^\dagger b b \rangle, \tag{S55}$$

$$\partial_t \langle b^\dagger \rangle = \left(\mathrm{i}\Delta - \frac{\gamma}{2}\right)\langle b^\dagger \rangle + \mathrm{i}\Omega \mathrm{e}^{-\mathrm{i}\theta}\langle b \rangle + \mathrm{i}U\langle b^\dagger b^\dagger b \rangle. \tag{S56}$$

The coupling of the first moments like $\langle b \rangle$ to the third moments like $\langle b^\dagger b b \rangle$, and thus to all odd-valued moments in general, implies an infinite set of equations. However, this infinite set of equations can be closed by employing the semi-classical approximation, whereby $\langle b^\dagger b b \rangle \simeq \langle b^\dagger \rangle \langle b \rangle^2$ and $\langle b^\dagger b^\dagger b \rangle \simeq \langle b^\dagger \rangle^2 \langle b \rangle$. In this regime, one may replace the mean value of the operator $\langle b \rangle$ by the general complex number

$$\langle b \rangle = B \mathrm{e}^{\mathrm{i}\beta}, \tag{S57}$$

of magnitude $B \geq 0$ and phase β. Substituting Eq. (S57) into Eq. (S55) yields the trivial solution $B = 0$ and the nontrivial solution

$$B = \sqrt{-\frac{\Omega}{U}\cos\left(\theta - 2\beta\right) - \frac{\Delta}{U}}, \qquad \sin\left(\theta - 2\beta\right) = \frac{\gamma}{2\Omega}, \tag{S58}$$

such that the mean population of the anharmonic oscillator in the semi-classical approximation $\langle b^\dagger b\rangle \simeq \langle b^\dagger\rangle\langle b\rangle$ is given by

$$\langle b^\dagger\rangle\langle b\rangle = \frac{-\Omega\cos(\theta-2\beta)-\Delta}{U} = \frac{1}{U}\left(\sqrt{\Omega^2-\left(\frac{\gamma}{2}\right)^2}-\Delta\right), \tag{S59}$$

which is given in the main text as Eq. (31) and is discussed around Fig. 6.

VIII. NONZERO TEMPERATURE

The open quantum system considered above (and in the main text) was treated at zero temperature. However, if the dissipation is due to a thermal bath, the quantum master equation of Eq. (S10) should be upgraded to the more general form

$$\partial_t\rho = \mathrm{i}[\rho,\hat{H}] + \frac{\gamma}{2}\left(1+N_\beta\right)\left(2b\rho b^\dagger - b^\dagger b\rho - \rho b^\dagger b\right) + \frac{\gamma}{2}N_\beta\left(2b^\dagger\rho b - bb^\dagger\rho - \rho bb^\dagger\right), \tag{S60}$$

where the Hamiltonian $\hat{H}$ is given by Eq. (S4), and where the number of excitations N_β in the thermal bath, at some effective inverse temperature β, is described by the Bose–Einstein distribution

$$N_\beta = \frac{1}{\mathrm{e}^{\beta\omega_0}-1}. \tag{S61}$$

Therefore, the quantum master equation used prior [cf. Eq. (S10)] is recovered in the zero temperature (or infinite inverse temperature) limit of $\lim_{\beta\to\infty} N_\beta = 0$. Otherwise, for some finite temperature the zero-temperature decay rate γ introduced in Eq. (S10) is renormalized to $\gamma(1+N_\beta)$, as in the second term appearing on the right-hand side of Eq. (S60). Furthermore, the exchange of energy between the oscillator and its environment becomes two-way at finite temperatures, modelled with the third term on the right-hand side of Eq. (S60), which describes the system being pumped at some lower gain rate γN_β. Crucially, the nonzero temperature master equation of Eq. (S60) does not change the location of the exceptional point away from $\Omega = \Delta$ or the critical driving amplitude away from Ω_c, as we now discuss.

A. Robustness of the exceptional point

The equation of motion for the first moments of the system at nonzero temperature follows from Eq. (S60) in a similar manner as to the calculation of Eq. (5) in the main text. Explicitly, we obtain

$$\mathrm{i}\partial_t\begin{pmatrix}\langle b\rangle\\ \langle b^\dagger\rangle\end{pmatrix} = \begin{pmatrix}\Delta-\mathrm{i}\frac{\gamma}{2} & \Omega\mathrm{e}^{\mathrm{i}\theta}\\ -\Omega\mathrm{e}^{-\mathrm{i}\theta} & -\Delta-\mathrm{i}\frac{\gamma}{2}\end{pmatrix}\begin{pmatrix}\langle b\rangle\\ \langle b^\dagger\rangle\end{pmatrix}, \tag{S62}$$

which is formally identical to Eq. (5) in the main text due to an exact cancellation of the temperature dependent terms (the extra loss as described by the rate $\gamma(1+N_\beta)$ is perfectly compensated by the extra gain at the rate γN_β coming into the system). Hence the first moments exceptional point at $\Omega = \Delta$ is indeed robust to nonzero temperatures. In the same way, the equation of motion for the second moments of the system is derivable from Eq. (S60) as

$$\mathrm{i}\partial_t\begin{pmatrix}\langle b^\dagger b\rangle\\ \langle bb\rangle\\ \langle b^\dagger b^\dagger\rangle\end{pmatrix} = \begin{pmatrix}\mathrm{i}N_\beta\gamma\\ \Omega\mathrm{e}^{\mathrm{i}\theta}\\ -\Omega\mathrm{e}^{-\mathrm{i}\theta}\end{pmatrix} + \begin{pmatrix}-\mathrm{i}\gamma & -\Omega\mathrm{e}^{-\mathrm{i}\theta} & \Omega\mathrm{e}^{\mathrm{i}\theta}\\ 2\Omega\mathrm{e}^{\mathrm{i}\theta} & 2\Delta-\mathrm{i}\gamma & 0\\ -2\Omega\mathrm{e}^{-\mathrm{i}\theta} & 0 & -2\Delta-\mathrm{i}\gamma\end{pmatrix}\begin{pmatrix}\langle b^\dagger b\rangle\\ \langle bb\rangle\\ \langle b^\dagger b^\dagger\rangle\end{pmatrix}. \tag{S63}$$

Notably, the 3×3 dynamical matrix inside Eq. (S63) is N_β-independent, and hence perfectly agrees with the form of the zero-temperature dynamical matrix M as defined by Eq. (12) in the main text. This ensures that the second moments exceptional point at $\Omega = \Delta$ is again unmoved despite the nonzero temperature of the environment, although the first (source) term on the right-hand side of Eq. (S63) does pick up a N_β dependence. For completeness, the same behaviour arises in the equation of

motion for the fourth moments, as follows [cf. Eq. (S34)]

$$
\mathrm{i}\partial_t \begin{pmatrix} \langle b^\dagger b\rangle \\ \langle bb\rangle \\ \langle b^\dagger b^\dagger\rangle \\ \langle b^\dagger b^\dagger bb\rangle \\ \langle bbbb\rangle \\ \langle b^\dagger b^\dagger b^\dagger b^\dagger\rangle \\ \langle b^\dagger bbb\rangle \\ \langle b^\dagger b^\dagger b^\dagger b\rangle \end{pmatrix} = \begin{pmatrix} \mathrm{i}N_\beta\gamma \\ -\mathrm{i}\Omega \mathrm{e}^{\mathrm{i}\theta} \\ \mathrm{i}\Omega\mathrm{e}^{-\mathrm{i}\theta} \\ 0 \\ 0 \\ 0 \\ 0 \\ 0 \end{pmatrix}
+ \begin{pmatrix}
-\mathrm{i}\gamma & -\Omega\mathrm{e}^{-\mathrm{i}\theta} & \Omega\mathrm{e}^{\mathrm{i}\theta} & 0 & 0 & 0 & 0 & 0 \\
2\Omega\mathrm{e}^{\mathrm{i}\theta} & 2\Delta-\mathrm{i}\gamma & 0 & 0 & 0 & 0 & 0 & 0 \\
-2\Omega\mathrm{e}^{-\mathrm{i}\theta} & 0 & -2\Delta-\mathrm{i}\gamma & 0 & 0 & 0 & 0 & 0 \\
4\mathrm{i}N_\beta\gamma & -\Omega\mathrm{e}^{-\mathrm{i}\theta} & \Omega\mathrm{e}^{\mathrm{i}\theta} & -2\mathrm{i}\gamma & 0 & 0 & -2\Omega\mathrm{e}^{-\mathrm{i}\theta} & 2\Omega\mathrm{e}^{\mathrm{i}\theta} \\
0 & 6\Omega\mathrm{e}^{\mathrm{i}\theta} & 0 & 0 & 4\Delta-2\mathrm{i}\gamma & 0 & 4\Omega\mathrm{e}^{\mathrm{i}\theta} & 0 \\
0 & 0 & -6\Omega\mathrm{e}^{-\mathrm{i}\theta} & 0 & 0 & -4\Delta-2\mathrm{i}\gamma & 0 & -4\Omega\mathrm{e}^{-\mathrm{i}\theta} \\
3\Omega\mathrm{e}^{\mathrm{i}\theta} & 3\mathrm{i}N_\beta\gamma & 0 & 3\Omega\mathrm{e}^{\mathrm{i}\theta} & -\Omega\mathrm{e}^{-\mathrm{i}\theta} & 0 & 2\Delta-2\mathrm{i}\gamma & 0 \\
-3\Omega\mathrm{e}^{-\mathrm{i}\theta} & 0 & 3\mathrm{i}N_\beta\gamma & -3\Omega\mathrm{e}^{-\mathrm{i}\theta} & 0 & \Omega\mathrm{e}^{\mathrm{i}\theta} & 0 & -2\Delta-2\mathrm{i}\gamma
\end{pmatrix}
\begin{pmatrix} \langle b^\dagger b\rangle \\ \langle bb\rangle \\ \langle b^\dagger b^\dagger\rangle \\ \langle b^\dagger b^\dagger bb\rangle \\ \langle bbbb\rangle \\ \langle b^\dagger b^\dagger b^\dagger b^\dagger\rangle \\ \langle b^\dagger bbb\rangle \\ \langle b^\dagger b^\dagger b^\dagger b\rangle \end{pmatrix}, \tag{S64}
$$

where it is again only the first (driving) term which captures nonzero temperature effects thanks to an explicit N_β dependence.

B. Some auxiliary results

Populations. The nature of the nonzero temperature dynamical equations, especially as defined in Eq. (S62) and Eq. (S63), means that the exceptional point physics of interest to us is essentially unchanged from the results quoted in the main text. However, the appearance of the temperature-dependent quantity N_β in the driving term of Eq. (S63) means that the steady state population of the parametrically-driven harmonic oscillator in a thermal bath is

$$
n(\infty) = \frac{\frac{\Omega^2}{2} + N_\beta\left[\Delta^2 + \left(\frac{\gamma}{2}\right)^2\right]}{\Delta^2 + \left(\frac{\gamma}{2}\right)^2 - \Omega^2}. \tag{S65}
$$

This result indeed reduces to the zero-temperature expression of Eq. (15) in the main text in the limit of no excitations in the thermal reservoir, $N_\beta \to 0$. Importantly, the critical driving amplitude Ω_{c} [cf. Eq. (16) in the main text] arising from the zero of the denominator of Eq. (S65) is temperature independent, joining the exceptional point as a robust feature of the main text model.

Squeezing. As well as Eq. (S65), the steady state solution of Eq. (S63) leads to the following temperature-dependent steady state correlators for the counter-rotating terms bb and $b^\dagger b^\dagger$ [cf. Eq. (S38)]

$$
\lim_{t\to\infty}\langle bb\rangle = -\left(N_\beta + \tfrac{1}{2}\right)\Omega\mathrm{e}^{\mathrm{i}\theta}\frac{\Delta + \mathrm{i}\frac{\gamma}{2}}{\Delta^2 + \left(\frac{\gamma}{2}\right)^2 - \Omega^2}, \qquad \lim_{t\to\infty}\langle b^\dagger b^\dagger\rangle = \lim_{t\to\infty}\langle bb\rangle^*. \tag{S66}
$$

These quantities enter the steady state quadrature variances, $\lim_{t\to\infty}\sigma_X^2$ and $\lim_{t\to\infty}\sigma_P^2$, as follows from Eq. (S18) and the formal variance definitions $\sigma_X^2 = \langle \hat{X}^2\rangle - \langle\hat{X}\rangle^2$ and $\sigma_P^2 = \langle\hat{P}^2\rangle - \langle\hat{P}\rangle^2$, like so

$$
\lim_{t\to\infty}\sigma_X^2 = \left(N_\beta + \tfrac{1}{2}\right)\frac{\Omega_{\mathrm{c}}^2 - \Omega\Delta}{\Omega_{\mathrm{c}}^2 - \Omega^2}, \qquad \lim_{t\to\infty}\sigma_P^2 = \left(N_\beta + \tfrac{1}{2}\right)\frac{\Omega_{\mathrm{c}}^2 + \Omega\Delta}{\Omega_{\mathrm{c}}^2 - \Omega^2}, \tag{S67}
$$

which reduce to the zero-temperature results, given in Eq. (29) in the main text, when $N_\beta \to 0$. The key impact of temperature is to modify the values of the driving amplitude Ω for which quantum squeezing, as defined by the inequality $\lim_{t\to\infty}\sigma_X^2 < 1/2$, occurs. It was shown in the main text that at zero temperature ($N_\beta \to 0$) steady state squeezing manifests for driving amplitudes below the exceptional point, since the limiting case of $\lim_{t\to\infty}\sigma_X^2 = 1/2$ is met at $\Omega = \Delta$. In the nonzero temperature case, as described by Eq. (S67), squeezing is instead seen to occur for a range of drivings Ω between the two bounds $\Omega_-^{\mathrm{squeeze}}$ and $\Omega_+^{\mathrm{squeeze}}$, where

$$
\Omega_\pm^{\mathrm{squeeze}} = \left(N_\beta + \tfrac{1}{2}\right)\Delta \pm \sqrt{\left(N_\beta + \tfrac{1}{2}\right)^2\Delta^2 - 2N_\beta\Omega_{\mathrm{c}}^2}. \tag{S68}
$$

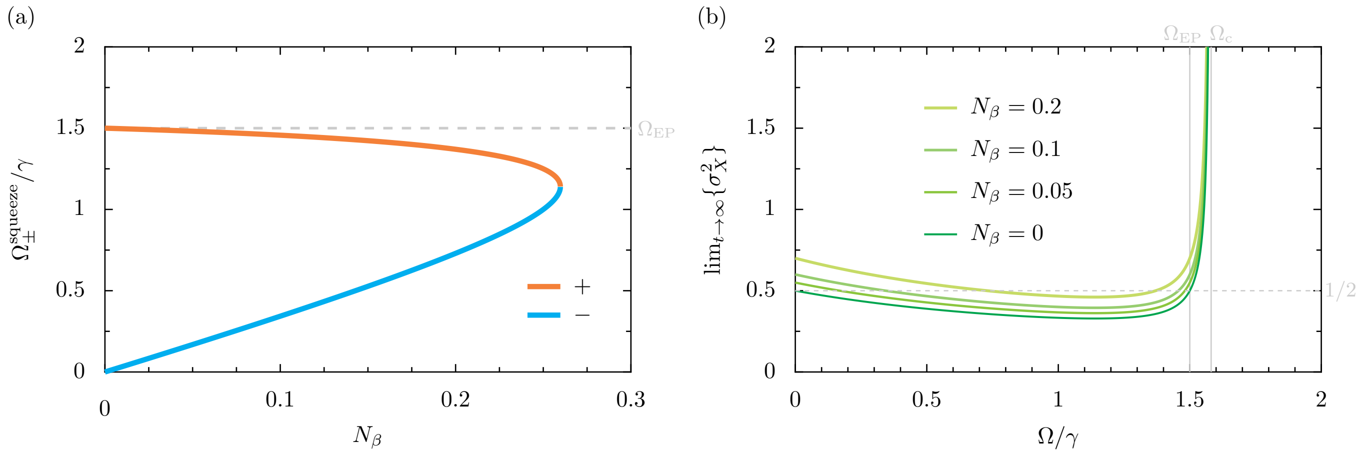

FIG. S7. **Squeezing of the parametric driven-dissipative oscillator.** Panel (a): the two bounds $\Omega_{\pm}^{\text{squeeze}}$ between which squeezing is possible, in units of the decay rate γ, as a function of the mean number of excitations in the thermal bath N_β [cf. Eq. (S68)]. Dashed line: the exceptional point at $\Omega_{\text{EP}} = \Delta$. Panel (b): the steady state quadrature variance $\lim_{t\to\infty}\{\sigma_X^2\}$ as a function of the driving amplitude Ω. We show results for increasing values of N_β with increasingly light lines. Horizontal line: the Robertson-Schrödinger minimum uncertainty of $1/2$ as a guide for the eye. Vertical lines: Ω_{EP} and the critical driving strength $\Omega_{\text{c}} = \sqrt{\Delta^2 + (\gamma/2)^2}$ as guides for the eye. In the figure, we consider the detuning $\Delta = 3\gamma/2$ so that $\Omega_{\text{EP}} = 3\gamma/2$ and $\Omega_{\text{c}} = \sqrt{5/2}\gamma \simeq 1.58\gamma$. Panel (b) extends the results of Fig. 5 (e) from the main text.

The two bounds in Eq. (S68) recover the zero-temperature case, since $\lim_{N_\beta\to 0}\Omega_{-}^{\text{squeeze}} = 0$ and $\lim_{N_\beta\to 0}\Omega_{+}^{\text{squeeze}} = \Delta$. For finite temperatures, Eq. (S68) defines a range of driving amplitudes Ω associated with quantum squeezing, as is suggested in Fig. S7 (a). In this panel, it can be seen that the exceptional point only coincides with a boundary value at zero temperature, and that at sufficiently high temperatures (corresponding to $N_\beta \simeq 0.26$ for the considered case of $\Delta = 3\gamma/2$) no squeezing is possible. The explicit relationship between the steady state quadrature variance $\lim_{t\to\infty}\{\sigma_X^2\}$ and the driving amplitude Ω is displayed in Fig. S7 (b), where the consistent asymptote at Ω_{c} and the shift in the range of drivings Ω permitting squeezing (for increasing values of N_β) are the most noticeable features.

Second-order coherence. The intensity coherence at zero time delay ($\tau = 0$), that is $g^{(2)}(0)$ as defined in Eq. (S42), is computable from the steady state solution of Eq. (S64) along with the mean oscillator population of Eq. (S65). We find the analytic expression

$$g^{(2)}(0) = 2 + \left(N_\beta + \tfrac{1}{2}\right)^2 \frac{\Omega^2\left[\Delta^2 + \left(\frac{\gamma}{2}\right)^2\right]}{\left(\frac{\Omega^2}{2} + N_\beta\left[\Delta^2 + \left(\frac{\gamma}{2}\right)^2\right]\right)^2}. \tag{S69}$$

Interestingly, the order one takes limits is important for this measure of coherence. As described in the main text around Eq. (25), by taking the zero-temperature limit ($N_\beta \to 0$) first with Eq. (S69) one finds

$$\lim_{N_\beta\to 0} g^{(2)}(0) = 2 + \frac{\Delta^2 + \left(\frac{\gamma}{2}\right)^2}{\Omega^2}, \tag{S70}$$

which suggests a divergence with vanishing driving amplitude Ω. However, if one instead takes the $\Omega \to 0$ limit first with Eq. (S69) then the result is the more familiar

$$\lim_{\Omega\to 0} g^{(2)}(0) = 2, \tag{S71}$$

which clearly holds for all temperatures since N_β no longer appears. In any case, such subtleties do not effect the exceptional point physics primarily discussed in the main text.

References

[S1] H.-P. Breuer and F. Petruccione, *The Theory of Open Quantum Systems* (Oxford University Press, Oxford, 2002).
[S2] C. Gardiner and P. Zoller, *The Quantum World of Ultra-Cold Atoms and Light, Book I: Foundations of Quantum Optics* (Imperial College Press, London, 2014).
